# Cyclin-Dependent Kinase Synthetic Lethality Partners in DNA Damage Response

**DOI:** 10.3390/ijms23073555

**Published:** 2022-03-24

**Authors:** Mateusz Kciuk, Adrianna Gielecińska, Somdutt Mujwar, Mariusz Mojzych, Renata Kontek

**Affiliations:** 1 Department of Molecular Biotechnology and Genetics, University of Lodz, Banacha 12/16, 90-237 Lodz, Poland; adrianna.gielecinska@edu.uni.lodz.pl (A.G.); renata.kontek@biol.uni.lodz.pl (R.K.); 2 Doctoral School of Exact and Natural Sciences, University of Lodz, Banacha Street 12/16, 90-237 Lodz, Poland; 3 M.M. College of Pharmacy, Maharishi Markandeshwar (Deemed to be University) Mullana, Ambala 133207, India; somduttmujwar@gmail.com; 4 Department of Chemistry, Siedlce University of Natural Sciences and Humanities, 3 Maja 54, 08-110 Siedlce, Poland; mmojzych@yahoo.com

**Keywords:** cyclin-dependent kinase (CDK), DNA damage response (DDR), inhibitor, MYC oncogene, poly (ADP-ribose) polymerase 1 (PARP-1), synthetic lethality

## Abstract

Cyclin-dependent kinases (CDKs) are pivotal mediators and effectors of the DNA damage response (DDR) that regulate both the pathway components and proteins involved in repair processes. Synthetic lethality (SL) describes a situation in which two genes are linked in such a way that the lack of functioning of just one maintains cell viability, while depletion of both triggers cell death. Synthetic lethal interactions involving CDKs are now emerging, and this can be used to selectively target tumor cells with DNA repair defects. In this review, SL interactions of CDKs with protooncogene products MYC, poly (ADP-ribose) polymerase (PARP-1), and cellular tumor antigen p53 (TP53) are discussed. The individual roles of each of the SL partners in DDR are described.

## 1. Introduction

Cyclin-dependent kinase (CDK) family members are essential components of numerous signaling pathways that govern transcription and cell-cycle progression. CDKs developed as a way to regulate the activity of cells in response to diverse cellular stimuli. Their role in the regulation of signaling pathways ensures that each cell duplicates its DNA precisely and that the DNA is evenly divided into two daughter cells. Moreover, CDKs play a crucial role in the cell cycle arrest upon DNA damage. Cancer cells characterized by uncontrolled proliferation show disturbances in cell cycle components, and a great deal of effort has been given to the development of anti-CDK agents [1]. Recently the role of CDKs in DNA damage response (DDR) has emerged, and many synthetic lethality partners of CDKs were established, as discussed in this review.

The phenomenon of synthetic lethality (SL) was first observed in fruit flies (*Drosophila pseudoobscura*) and was found to occur when two genes are linked in such a way that the lack of functioning of just one maintains cell viability while depletion of both genes causes cell death. In its simplest and most desired form, this would kill cancer cells while leaving healthy ones unharmed [2,3]. Clinically, synthetic lethality has three primary advantages: (a) treatment strategy might address most cancer mutations, (b) SL makes it easier to identify patients who are responding to treatment because of its selective nature, (c) it may be used to improve chemotherapeutic drug combination therapy allowing reduction of drug dosages, at the same time lowering their side effects [3].

## 2. CDK/MYC and DDR

### 2.1. MYC and DDR

The MYC oncoprotein’s remarkable history spans three decades of fast-paced scientific research. Many types of human cancer are fueled by the MYC oncogene. MYC is a member of a protein family consisting of C-MYC, N-MYC, and L-MYC, and its expression is tightly controlled by several transcriptional regulatory elements located in the proximal promoter regions of the *MYC* gene. MYC, together with MAX protein, forms a dimeric transcription factor that binds to sophisticated DNA response elements (E-boxes) and governs the transcription of many downstream genes associated with proliferation [4].

**Figure 1:**
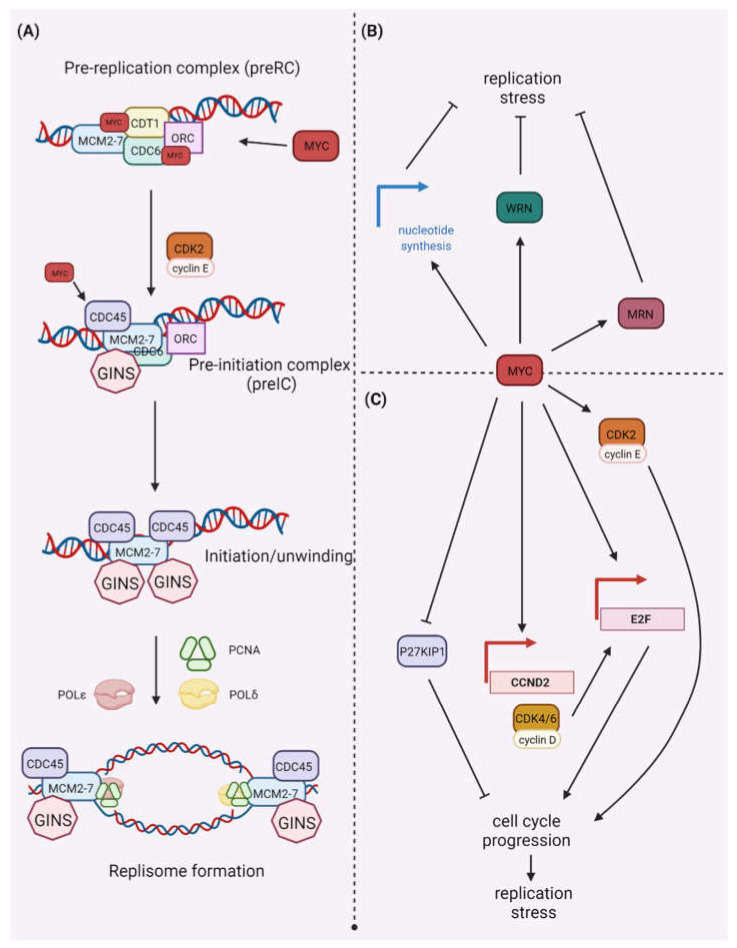
MYC’s role in (**A**) replication and (**B**,**C**) replication stress. (**A**) The origin recognition complex (ORC) binds to the replication origin site and recruits the DNA replication factor Cdt1 (CDT1) and the cell division control protein 6 homolog (CDC6), which aid in the loading of the DNA replication licensing factor (MCM2-7) helicases to form the pre-replicative complex (pre-RC). The pre-RC is phosphorylated by CDK2 and protein DBF4 homolog A/B (DBF4/DRF1)-dependent cell division cycle 7-related protein kinase (CDC7), permitting recruitment of cell division control protein 45 homolog (CDC45) and the DNA replication complex GINS. During origin licensing, MYC physically interacts with pre-RC components such as ORC, CDC6, CDT1, and MCMs. MYC also promotes ORI activation by boosting CDC45 chromatin binding, a replication factor required for DNA replication initiation. (**B**) When the pRB-E2F pathway is disrupted, MYC protein can alleviate replication stress by enhancing nucleotide biosynthesis pathways, increasing the purine and pyrimidine pool, and relieving replication stress caused by high DNA synthesis rates. MYC proteins also directly enhance the expression of specific replication proteins, including the WRN helicase, which resolves odd replication intermediates, and the MRN nuclease. (**C**) Activation of MYC causes DNA damage and genomic instability by impairing DNA replication dynamics, causing collisions between replication and transcription machinery. The MYC-induced replication stress indirectly activates the cyclin E/CDK2 complex by inducing *CCND2* gene expression, inactivating the CDK inhibitor P27KIP1, or stimulating E2F–mediated gene expression. Created with BioRender.com (accessed on 1 February 2022).

As a transcription factor, MYC’s capacity to stimulate cellular proliferation is due to its ability to directly control the expression of a vast variety of cellular pathway components that are involved in the advancement of the S-phase [5]. Furthermore, MYC can localize to active DNA replication sites, implying that MYC plays a more direct role in directing S-phase progression via activation or promotion of the formation of replication complexes [6]. Strong proliferative responses by oncogenes are usually accompanied by increased DDR activation. This link is strongest in the early phases of tumor growth, supporting the concept that oncogene-induced DDR is a response with tumor-suppressive properties [7]. However, while unconstrained, MYC activation can cause replication stress. DNA replication encompasses generally two stages: licensing and initiation. Thousands of replication origins are formed across the genome, ensuring just one replication per cell cycle. The origin recognition complex (ORC) attaches to the replication origin site and recruits DNA replication factor Cdt1 (CDT1) and cell division control protein 6 homolog (CDC6), which help load the DNA replication licensing factor (MCM2-7) helicases to form the pre-replicative complex (pre-RC). The pre-RC is phosphorylated by CDK2 and cell division cycle 7-related protein kinase (CDC7), permitting recruitment of cell division control protein 45 homolog (CDC45) and the DNA replication complex GINS, subsequently activating the replicative helicase CMG (CDC45-MCM-GINS) complex (Figure 1). 

Then, a replication bubble forms, and replication forks spread in both directions. Normally, an excess of origins is licensed, and only a few are used, leaving the others as backups [8]. Oncogene activation may disrupt normal replication, putting genomic areas with DNA secondary structures in danger. Additionally, collisions between replication and transcription machinery might generate DNA replication stress. A majority of these conflicts occur in genomic regions encoding large genes that need more than one round of replication. This results in an increase in the number of stalled replication forks and the formation of double strand breaks (DSBs) [9]. MYC-induced replication stress causes DNA damage and genomic instability during carcinogenesis. The topic of mechanisms of replication stress induction by oncogenes has been recently described [8,9]. 

Overexpression of c-MYC alters cellular metabolism, leading to increased reactive oxygen species (ROS) generation and oxidative DNA damage [10]. Additionally, activation of MYC causes DNA damage via direct influence on DNA replication dynamics [11,12]. MYC-induced replication stress interferes with origin firing and licensing. During origin licensing, MYC physically interacts with replication machinery components such as ORC, CDC6, CDT1, and MCMs. MYC also promotes ORI activation by boosting CDC45 chromatin binding that acts as a replication factor required for DNA replication initiation (Figure 1) [6,13]. However, the specifics of the MYC-mediated replication stress mechanism remain unknown. The MYC-induced replication stress indirectly activates the CDK2/cyclin E complex. MYC enhances progression through the cell cycle and boosts CDK2/cyclin E activity by inducing cyclin D2 expression, inactivating CDK inhibitor P27KIP1, or stimulating the expression of genes controlled by E2F transcription factor [14]. The effects of P27KIP1 in the suppression of the cell cycle are due to its ability to restrict the function of CDK2/cyclin E complexes. Furthermore, ataxia telangiectasia mutated (ATM) kinase phosphorylates P27KIP1 on serine 140 (Ser-140). This increases the stability of P27KIP1, enabling it to trigger cell cycle arrest. Additionally, P27 promotes the accumulation of RAD51 during double-strand break repair (DSBR), allowing efficient DNA repair [15]. MYC protein can combat replication stress. Many genes responsible for the control of proliferation and DNA replication are induced by the MYC transcription factor [16,17]. Enhancement of the purine and pyrimidine metabolism helps relieve replication stress caused by high DNA synthesis rates when the major pathway of cell division pRB-E2F is disrupted [18]. MYC protein also directly upregulates the expression of specific replication enzymes, including the Werner syndrome ATP-dependent helicase (WRN) [19,20], which resolves odd replication intermediates, or the MRN nuclease (MRE11/RAD50/NBS1) [21], which repairs DSBs and restarts collapsed replication forks. WRN, a RECQ DNA helicase, resolves topologically unfavorable DNA structures such as halted replication forks [22,23].

It is noteworthy that, as a crucial transcription factor, MYC regulates the expression of a wide range of DNA damage repair proteins, including the genes RAD51, X-ray repair cross-complementing protein 2/4 (XRCC2/4), breast cancer type 1 susceptibility protein 1/2 (BRCA1/2), DNA-dependent protein kinase catalytic subunit (DNA-PKcs), and X-ray repair cross-complementing protein 5 (KU70) [24]. MYC was shown to have an opposing role in DDR signaling by two distinct pathways—ATM/CHK2 (obstacle for malignant transformation) and ATR/CHK1 (tumor maintenance). Currently, the most well-characterized component of MYC-induced tumor suppression involves the tumor suppressor P19/ARF (ARF)/E3 ubiquitin-protein ligase MDM2 (MDM2)/TP53 pathway, which leads to the activation of an apoptotic response that is dependent on the TP53 protein [25,26,27]. When oncogenic stress occurs, MYC increases the levels of ARF, which in turn stabilizes and activates TP53 [28,29]. However, activated MYC can lead to genomic and chromosomal instability [30]. Recent research has identified ATM [31,32,33], protein phosphatase 1D (WIP1) [34], and histone acetyltransferase KAT5/TIP60 [35] as mediators of MYC-induced DDR, with the established role of ATM as a suppressor of the response to damage. In human malignancies, particularly B-cell lymphomas, MYC activation is linked to ATM inactivation [36]. Loss of ATM reduces DDR and MYC-induced apoptosis while increasing tumorigenesis. Of importance, ATM kinase may act on TP53 independently of ARF [31,33]. TIP60, a histone acetyltransferase (HAT) involved in the regulation of DNA repair and DDR via activation of ATM, showed similar results. NUA4-TIP60 complex is attracted to sites of DNA damage, and its primary role is to acetylate histone H2AX and H4, allowing changes in DNA architecture for efficient repair [37,38,39]. TIP60 appears to be essential for the effective induction of DDR by the MYC oncogene [35]. Many commonly used chemotherapeutic agents will not work on DDR-deficient tumors, as many of them require a functional DDR response. However, cancers lacking DDR effector activities may allow for pharmacological targeting of certain DDR branches that in normal cells are compensated by other pathways without negative effects on cell survival [33]. 

### 2.2. MYC Synthetic Lethality Partners

MYC pathway-specific small molecule inhibitors are scarce partially due to nuclear localization of the protein, as well as its vital physiological activities for the maintenance of normal tissues. High-throughput synthetic lethal screens reveal more than a hundred candidate genes that are potentially fatal to the cells showing disturbances in MYC function and expression [4,5,40]. These include regulators of MYC stability such as polo-like kinase 1 (PLK1), serine/threonine-protein kinase pim-1 (PIM-1), and aurora kinases A and B (AURKA and AURKB), cooperating transcriptional factors: MAX and zinc finger and BTB domain-containing protein 17 (MIZ1), DNA damage checkpoint regulators: CHK1, DNA-PKcs, ATR, antiapoptotic protein-myeloid leukemia cell differentiation protein (MCL1), regulators of mitochondrial translation for energy metabolism AMPK-related protein kinase (ARK5), or inosine-5-monophosphate dehydrogenase (IMPDH2), unfolded protein response (UPR) components including PRKR-like endoplasmic reticulum kinase (PERK), cyclic AMP-dependent transcription factor ATF4 (ATF4), serine/threonine-protein kinase/endoribonuclease IRE (IRE1α) and X-box-binding protein 1 (XBP1), and serine/threonine kinase mTOR-dependent protein synthesis pathway components [41,42,43,44]. MYC protein is strongly involved in the metabolic shift of cancer cells; thus, glycolysis and glutaminolysis inhibitors have also proven to be effective in tackling cancer cells via a conditional synthetic lethality approach. This matter was extensively discussed in the recent paper of Hsieh and Dang [45]. Here, we provide just a summary of their work. The idea that MYC increases gene expression is consistent with its ability to enhance the expression of housekeeping genes implicated in the control of metabolism and ribosome biosynthesis. Since practically all nucleated cells require these genes for proper function, accessibility for transcription factors is high. Moreover, genes encoding the proteins engaged in cellular metabolism are equipped with conventional MYC E-boxes, suggesting their control is dependent on MYC oncoprotein. MYC appears to trigger glycolytic enzyme gene expression in early G1 and regulates enzymes involved in glycolysis, including hexokinase 2 (HK2) and other glycolysis-related genes. Furthermore, MYC-binding sites overlap with hypoxia-inducible factor 1 (HIF-1) binding sites and promote anaerobic glycolysis as a strategy to adapt and survive in oxygen- and nutrient-depleted conditions. Oncogenic MYC overexpression results in changes in cell transcriptome that is distinct from that typically observed in cells with physiological MYC levels. Studies in human fibroblasts demonstrated that deletion of glucose metabolism genes encoding fructose-bisphosphate aldolase A (ALDOA) and pyruvate dehydrogenase kinase isoform 1 (PDK1), nucleotide metabolism proteins CTP synthase (CTPS), or nutrient transporters (neutral amino acid transporter A (SLC1A4) and ADP/ATP translocase 3 (SLC25A6)) caused synthetic lethality when MYC was overexpressed. A screen for the expanded MYC transcription factor network indicated that depletion of MLX-interacting protein (MONDOA) resulted in selective lethality of MYC-overexpressing cells. MONDOA was linked to glucose metabolism, hence the synthetic lethal screen was broadened to metabolic genes. Glutamine/glutamate transporters: neutral amino acid transporter B(0) (SLC1A5) and 4F2 cell-surface antigen heavy chain (SLC3A2); purine metabolism enzymes: phosphoribosylformylglycinamidine synthase (PFAS), cystathionine beta synthase (CBS), and mitochondrial transcription factor A (TFAM); glycolysis enzyme: β-enolase (ENO3), and lipogenesis enzymes: fatty acid synthase (FASN) and stearoyl-CoA desaturase (SCD) were identified as MYC synthetic lethality partners in the screen [45].

**Figure 2:**
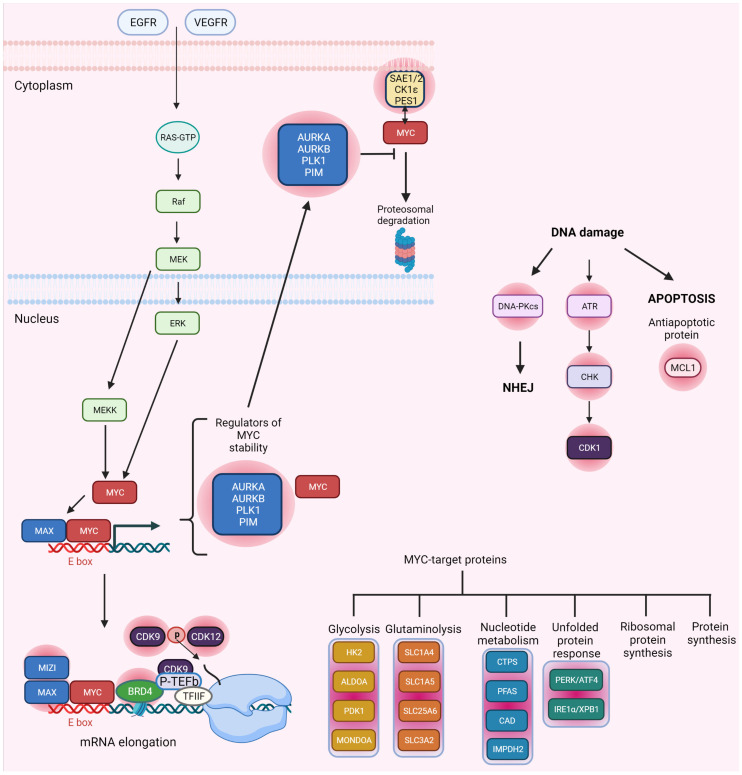
MYC-dependent control of gene expression and synthetic lethality partners of MYC. In response to mitogenic stimuli, the RAS-RAF-MEK-ERK pathway triggers MYC-dependent gene expression of enzymes involved in the control of glycolysis, glutaminolysis, nucleotide metabolism, unfolded protein response, general protein synthesis, and ribosomal protein synthesis, many of which were identified as synthetic lethality partners of MYC (indicated as pink shadows). For example, CDK9 and CDK12, which control the mRNA elongation by RNA polymerase II via phosphorylation of the CTD domain of the enzyme, were identified as MYC-SL partners. Regulators of MYC stability that prevent the proteasomal degradation of the protein, and four DDR components, namely ATR, CHK1, CDK1, and DNA-PKcs, also constitute a pool of MYC-SL partners. Based on [41,45,46]. Created with BioRender.com (accessed on 1 February 2022).

In addition, several other proteins not related to the previous classes of enzymes were identified. For example, lack of SUMO-activating enzyme subunit 1/2 (SAE1/2) resulted in widespread loss of SUMOylation, G2/M cell accumulation, aberrant mitotic spindle formation, and mitotic catastrophe. According to network analysis, SAE1 interacts with two other MYC-SL partners, SUMO-conjugating enzyme UBC9 (UBE2I) and E3 ubiquitin-protein ligase MDM2 (MDM2) involved in DDR through the TP53-MDM2 axis, which may explain why SAE1/2 knockdown is lethal. On the other hand, PES1, the human homolog of Pescadillo, involved in zebrafish embryonic development, may be a direct target of MYC transcriptional control. PES1 has a BRCT domain, which seems pivotal for the function of multiple DNA repair genes, including BRCA1. PES1’s BRCT domain may influence large chromatin domain folding, and the protein may be involved in rRNA synthesis. In neuroblastoma cells, its loss causes both cell death and differentiation. F-box/WD repeat-containing protein 7 (FBWX7) regulates the ubiquitin-dependent degradation of MYC. Deletion of FBW7 in cells with MYC overexpression may cause cell death [46]. A meta-analysis of primary neuroblastoma microarray data linked casein kinase I isoform epsilon (CK1ε) expression to MYCN amplification and poor prognosis. It was also shown that reduction of CK1ε expression diminishes the proliferation of neuroblastoma cell lines with enhanced MYC expression [47]. Synthetic lethality partners of MYC are shown in Figure 2.

### 2.3. MYC and CDK Synthetic Lethality

Survival of cells overexpressing MYC necessitates the presence of the CDK1 target protein baculoviral IAP repeat-containing protein 5 (BIRC5), which inhibits apoptosis. Therefore, inhibition of CDK1 may be used to target human malignancies in which overexpression of MYC occurs [48]. CDK1 inhibitors may be particularly effective against aggressive breast cancers that express high levels of MYC, as the selective inhibition of CDK1 with purvalanol A triggers the apoptotic response in triple-negative tumor xenografts [49]. The inactivation of CDK1 significantly increases apoptosis and decreases the viability of MYC-dependent cells. CDK1 inhibitors selectively increase apoptosis by upregulating the pro-apoptotic protein Bcl-2-like protein 11 (BIM), without affecting TP53. However, no such effects were observed with the use of CDK2 and CDK4/6 inhibitors [50]. This is not surprising given that CDK1 is the only CDK necessary for the progression of the cell cycle [51]. Moreover, CDK2 was identified as a synthetic lethality partner of N-MYC in neuroblastoma cells, as therapeutically feasible doses of roscovitine, a CDK inhibitor, caused N-MYC-dependent cell death, supporting the notion of synthetic lethality between the two proteins [52]. Cellular senescence and apoptosis are examples of tumor-suppressing responses triggered by activated oncogenes. In vivo, loss of CDK2 makes pancreatic cells and splenic B-cells more susceptible to MYC-induced senescence, which coincides with a delayed beginning of lymphoma in the latter [53]. It was found that inhibitors of bromodomain-containing protein 4 (BRD4) [54,55,56], CDK7 [57], and CDK9 [58,59] may repress MYC expression. MYC also works as a binding partner for transcription elongation factor (P-TEFb) encompassing CDK9 and recruits transcription complexes encompassing RNA polymerase II (RNAP-II) for enhanced transcription of MYC-dependent genes [59]. CDK7 and/or CDK9 inhibition significantly lowers MYC expression, resulting in changes in MYC-dependent gene expression profiles. Moreover, specific inhibitors of CDK7 and CDK9 have enhanced anticancer effects in MYC-driven malignancies, supporting their synthetic lethality relationship with MYC [57,60]. CDK9 inhibition by dinaciclib appears to be particularly successful in promoting the regression of aggressive MYC-driven lymphomas by selectively inhibiting key MYC targets such as MCL1 [59]. Moreover, CDK12 inhibition may induce the synthetic lethality phenomenon in MYC-dependent cancers [47,61]. In ovarian cancer cells, THZ1, a compound that inhibits CDK7, CDK12, and CDK13, dramatically reduces the activity of MYC. Notably, inhibiting MYC expression requires suppressing CDK7, CDK12, and CDK13 all at once rather than just targeting CDK7 [47,62].

## 3. CDK/TP53 and DDR

TP53 is a tumor suppressor protein engaged in many cellular processes, including response to DNA damage and apoptosis. Estimates suggest that *TP53* gene mutations are responsible for around half of all solid cancers. The DNA damage-induced posttranslational modifications of wild-type (wt) TP53 protein include phosphorylation of N-terminal serines by numerous kinases, including ATM, ATR, and DNA-PKcs, and acetylation of its core DNA binding region. Stabilized and activated wt TP53 triggers various pathways and responses, including apoptosis and cellular senescence. TP53 activates the expression of additional pro-apoptotic proteins such as BIM, apoptotic protease-activating factor 1 (APAF1), tumor necrosis factor receptor superfamily member 6 (FAS/CD95), and tumor necrosis factor receptor superfamily member 10 (TRAIL-R2/DR540). TP53 also interacts with the antiapoptotic protein BCL-xL, preventing it from sequestering BAX or Bcl-2 homologous antagonist/killer (BAK1) protein. TP53 is responsible for the regulation of ferroptosis and autophagic cell death. Additionally, TP53 promotes ferroptosis by inhibiting the cystine/glutamate transporter (SCL7A11) and controls the expression of the CDK inhibitor P21, and thereby the induction of cell arrest in the G1/S and G2/M checkpoints. In base excision repair (BER), DNA-(apurinic or apyrimidinic site) endonuclease (APE1) and TP53 interplay has been shown. The interaction of the tumor suppressor with the DNA POLβ may enhance the aforementioned type of DNA repair. TP53 has both transcription-dependent and independent roles in nucleotide excision repair (NER). TP53 interacts with DNA repair protein complementing XP-C cells (XPC), general transcription and DNA repair factor IIH helicase subunit XPB (XPB), and Cockayne syndrome protein B (CSB) and inhibits the general transcription and DNA repair factor IIH helicase subunit XPD (XPD/RAD3) and XPB DNA helicase. TP53 is also involved in DSBR, where it interacts with replication protein A (RPA), RAD54, BRCA1, BRCA2, bloom syndrome protein (BLM), and WRN. Furthermore, non-homologous end joining (NHEJ) is also controlled by TP53, but its role is unclear [63,64,65,66].

It was found that overexpression of CDK2 is critical for the G1/S checkpoint activation in DDR. Knocking down CDK2 in the HCT116 cell line lowers TP53 phosphorylation following incubation of cells with hydroxyurea [67]. The fact that CDK2 can alter the apoptotic signaling in immortalized epithelial cells (HaCaT) and lead to apoptosis of TP53-deficient HaCaT cells indicates the SL interaction between these two proteins. Furthermore, the inhibition of CDK2 cause a decrease in the phosphorylation at the RAC-alpha serine/threonine-protein kinase (AKT) on Ser-473/474 in the S/G2 phase [68]. Of note, CDK1/2 and phosphatidylinositol 4,5-bisphosphate 3-kinase (PI3K) are a pair of synthetic lethality partners and may provide new targets for anticancer drug discovery, especially in malignant gliomas [69]. Targeting of CDKs also can be useful in the therapy of aggressive triple-negative breast cancers (TNBC) with high metastatic capabilities. The G1 checkpoint is bypassed by most TNBC cells harboring *TP53* mutations, allowing them to proceed through the cell cycle despite DNA damage. In the G2-M cell-cycle phase, roscovitine treatment halts TNBC cells, making them vulnerable to DNA damage. When compared to doxorubicin alone, combination therapy with a CDK inhibitor increases the frequency of DSBs while decreasing the recruitment of proteins involved in homologous recombination (HR). The combination of drugs decreases tumor burden and improves overall survival in xenograft studies compared to single drugs or concurrent treatments. To achieve combination-induced cytotoxicity, the TP53 pathway must be shut down, which makes TP53 a potential biomarker for treatment response in TNBC [70]. In the TP53-proficient HCT116 cell line, both activation and inhibition of TP53 and CDK7 exhibit SL properties. Apoptotic cell death can be induced by pretreatment of cells with 5-FU or nutlin-3, and then treatment with the CDK7 inhibitor (THZ1 or YKL-1-116). The TP53 transcriptional target-death receptor (DR5) is required for the induction of apoptosis. A concurrent decrease in expression of MDM2 and P21 is observed [71]. 

## 4. CDK/PARP and DDR

Poly PARPs are a family of related nuclear enzymes that transfer ADP-ribose to various target proteins. PAPR-1 is involved in the addition of branched poly(ADP-ribose) polymers (PAR) to DNA targets, histones, and DNA repair proteins. Histone modification opens the chromatin structure, allowing repair enzymes access to the damaged DNA regions. The PARP-1-DNA interface is only transient due to electrostatic interactions. PARG and ARH3 inhibit PARylation by the digestion of the PAR polymers of PARP-1 targets [72]. Many scientists have previously evaluated the role of PARP in genomic integrity, DNA repair, and PARP inhibition in cancer, and their findings are summarized elsewhere [73,74]. 

Clinically approved PARP inhibitors include olaparib, rucaparib, and talazoparib. New PARP inhibitors with more DNA trapping potential, superior safety profiles, or enhanced combination therapies could be introduced to the clinical setting in the future [75]. Synthetic lethal anticancer treatments including PARP inhibitors can target tumor cells with specific HR defects. This approach is supported by single-agent antitumor efficacy and the wide therapeutic index of PARP inhibitors in BRCA1 and BRCA2 mutation carriers with advanced malignancies. Investigations have shown that BRCA1 and BRCA2 mutant cells can become resistant to PARP inhibitors by accumulating secondary mutations that restore a partially functioning gene. This may limit the treatment efficiency [76]. New evidence suggests that PARP inhibitors may be useful in sporadic malignancies with HR abnormalities, indicating a much broader utility for PARP inhibitors [73,77]. 

It has been demonstrated that depletion of CDK1 or pharmacological inhibition of CDK1 increases the susceptibility of BRCA-positive breast cancer to PARP inhibitors. CDK1 is required for the HR repair pathway; therefore, blocking it can mimic BRCA1 mutation and boost TNBC cell sensitivity to PARP inhibitors by 100-fold [78,79]. Multiple myeloma cells exhibit chromosomal instability and widespread DNA damage, implying faulty DNA repair. Malignant melanoma cells also display dysregulation in CDK activities. Dinaciclib was shown to inhibit HR repair and sensitize cells to the PARP1/2 inhibitor ABT-888. Dinaciclib reduces the formation of ABT-888-induced BRCA1 and RAD51 foci while at the same time increasing γH2AX foci formation [80]. Dinaciclib, in addition to its capacity to inhibit CDK1 and CDK2, also exhibits anti-CDK12 activity and reduces the expression of BRCA1, BRCA2, and RAD51, making BRCA-wild type triple-negative breast cancer more sensitive to PARP inhibition [81]. Ro-3306 works as a CDK1-specific inhibitor that causes cancer cells to enter apoptosis by arresting the cell cycle at the G2 to M transition. It has been demonstrated to disrupt HR repair and to make BRCA-positive breast cancer more sensitive to PARP inhibitors [78]. Recent studies reveal that CDK12 suppresses premature poly-A cleavage and impacts long-chain (>45 kb) mRNA extension, causing aberrant HR repair gene expression [82].

## 5. Conclusions

**Figure 3:**
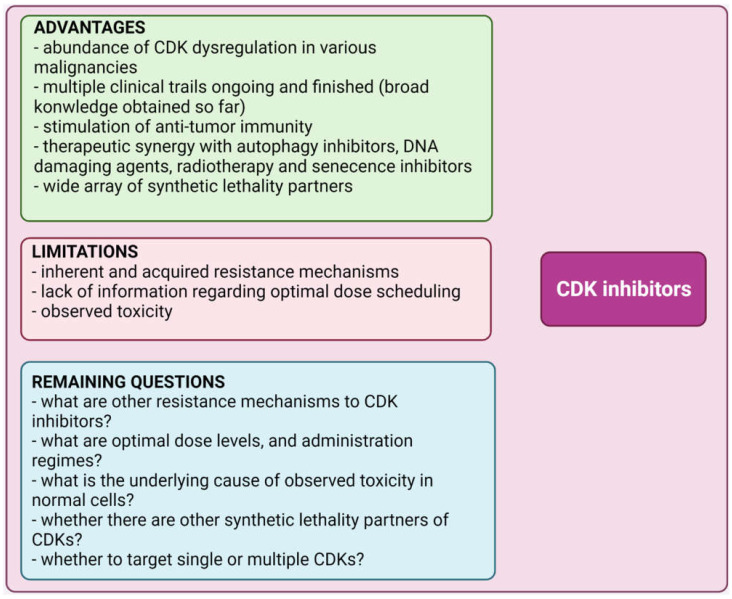
Advantages, limitations and remaining questions regarding use of cyclin-dependent kinase (CDK) inhibitors. Created with BioRender.com (accessed on 1 February 2022).

Despite continuous progress in anticancer drug development, there is still no effective anti-cancer treatment. This may be attributed to the side effects exhibited by non-selective agents in normal cells. Combining more than one agent can help to alleviate these obstacles. In recent years, advances in precision medicine and progress in the field of DNA damage response (DDR) and DNA repair have allowed the identification of many proteins with pivotal functions for cell survival. Given the important roles of MYC, PARP1, and TP53 in the DDR pathways, these enzymes have attracted considerable attention. More recently, a more sophisticated role of CDKs in DDR has emerged [83]. CDK inhibitors have been extensively examined in clinical trials [84,85]. Inhibition of CDK offers many advantages but also implies several limitations, as described in Figure 3.

Intimate influences of CDK7, CDK9, and CDK12/13 on transcriptional regulation and DDR have recently been established. This suggests new therapeutic approaches to cancer treatment based on synthetic lethality incorporating CDK-directed inhibitors [86,87,88,89]. In terms of opportunities for rapid clinical application of CDK inhibitors, the revelation that CDKs can stimulate anti-tumor immunity is particularly important. For example, palbociclib or abemaciclib increases interferon and major histocompatibility complex (MHC) expression, enhancing the antigen-presenting ability of tumor cells [85]. Cancer immunotherapy has shown promising outcomes in recent years, and this is expected to continue in the upcoming years [90]. Potential inherent and acquired resistance mechanisms have been extensively studied in recent years to extend the benefit of CDK inhibitors [85]. The effect of CDK inhibitors on normal cells and the tumor microenvironment is a critical issue considering their clinical utility. In normal cells, targeting CDKs may disrupt upstream cell cycle genes such as sirtuins, leading to cellular senescence and premature aging [91].

Many common chemotherapy methods will not work on DDR-deficient tumors, as many of them require a functional DDR response. However, in malignancies that lack DDR effector functions, pharmaceutical targeting of specific DDR branches that are sometimes complemented in healthy cells may effectively lead to their death. The synthetic lethality approach with the lowered potential of resistance development and diminished toxic effects on normal cells is now an emerging trend in cancer treatment, and new synthetic lethality screens will allow the identification of novel synthetic lethality partners of CDKs. Beyond the simple synthetic lethal interactions involving one or even two pathways, more complex synthetic lethal interactions involving CDK synthetic lethality partners that span multitude pathways can be discovered in the future [92]. This area of research may lead to the development of new therapies for the effective treatment of cancer.

## Abbreviations

AKT	RAC-alpha serine/threonine-protein kinase
ALDOA	Fructose-bisphosphate aldolase A
APAF1	Apoptotic protease-activating factor 1
APE1	DNA-(apurinic or apyrimidinic site) endonuclease
ARF	Tumor suppressor ARF
ARH3	ADP-ribose glycohydrolase ARH3
ARK5	AMPK-related protein kinase
ATF4	Cyclic AMP-dependent transcription factor ATF4
ATM	Ataxia telangiectasia mutated
ATR	Ataxia telangiectasia and Rad3-related protein
AURKA/AURKB	Aurora kinases A and B
BAK1	Bcl-2 homologous antagonist/killer
BAX	Pro-apoptotic protein BAX
BER	Base excision repair
BIM	Bcl-2-like protein 11
BIRC5	Baculoviral IAP repeat-containing protein 5
BLM	Bloom syndrome protein
BRCA1/2	Breast cancer type 1 susceptibility protein ½
BRD4	Bromodomain-containing protein 4
CBS	Cystathionine beta synthase
CDC45	Cell division control protein 45 homolog
CDC6	Cell division control protein 6 homolog
CDC7	Cell division cycle 7-related protein kinase
CDK	Cyclin-dependent kinase
CDKi	Cyclin-dependent kinase inhibitors
CDT1	DNA replication factor Cdt1
CHK1/2	Checkpoint kinase ½
CK1ε	Casein kinase I isoform epsilon
CMG	Replicative helicase CMG
CSB	Cockayne syndrome protein B
CTPS	CTP synthase 1
DDR	DNA damage response
DNA-PKcs	DNA-dependent protein kinase catalytic subunit
DSBR	Double-strand break repair
DSBs	Double-strand breaks
ENO3	β-enolase
FAS/CD95	Tumor necrosis factor receptor superfamily member 6
FASN	Fatty acid synthase
FBXW7	F-box/WD repeat-containing protein 7
HAT	Histone acetyltransferase
HIF-1	Hypoxia-inducible factor 1
HK2	Hexokinase 2
HR	Homologous recombination
IMPDH2	Inosine-5-monophosphate dehydrogenase
IRE1α	Serine/threonine-protein kinase/endoribonuclease IRE
KAT5/TIP60	Histone acetyltransferase KAT5/TIP60
KU70/80	X-ray repair cross-complementing protein 5/6
MAPK	Mitogen-activated protein kinases
MCL1	Myeloid leukemia cell differentiation protein
MCM2-7	DNA replication licensing factor MCM2-7
MDM2	E3 ubiquitin-protein ligase MDM2
MHC	Major histocompatibility complex
MIZ1	Zinc finger and BTB domain-containing protein 17
MONDOA	MLX-interacting protein
MRE11	Double-strand break repair protein MRE11
NAD	Nicotinamide adenine dinucleotide
NBS1	Nibrin
NER	Nucleotide excision repair
NHEJ	Non-homologous end-joining
ORC	Origin recognition complex
P27KIP1	Cyclin-dependent kinase inhibitor 1B
PAR	Poly (ADP-ribose) polymers
PARG	Poly (ADP-ribose) glycohydrolase
PARPs	Poly [ADP-ribose] polymerases
PD-1	Programmed cell death protein 1
PDK1	Pyruvate dehydrogenase kinase isoform 1
PERK	PRKR-like endoplasmic reticulum kinase
PES1	Pescadillo homolog
PFAS	Phosphoribosylformylglycinamidine synthase
PI3K	Phosphatidylinositol 4,5-bisphosphate 3-kinase
PIM-1	Serine/threonine-protein kinase pim-1
PLK1	Polo-like kinase 1
pRB	Retinoblastoma protein
Pre-RC	Pre-replicative complex
PTEF-b	Transcription elongation factor
RAD51	DNA repair protein RAD51 homolog
RNAP-II	RNA polymerase II
ROS	Reactive oxygen species
RPA	Replication protein A
SAE1/2	SUMO-activating enzyme subunit ½
SCD	Stearoyl-CoA desaturase
SCL7A11	Cystine/glutamate transporter
SLC1A4	Neutral amino acid transporter A
SLC1A5	Neutral amino acid transporter B(0)
SLC25A6	ADP/ATP translocase 3
SLC3A2	4F2 cell-surface antigen heavy chain
SSBR	Single-strand break repair
SSBs	Single-strand breaks
SsDNA	Single-stranded DNA
TFAM	Mitochondrial transcription factor A
TNBC	Triple-negative breast cancer
TP53	Cellular tumor antigen p53
TRAIL R2/DR540	Tumor necrosis factor receptor superfamily member 10
UBE2I	SUMO-conjugating enzyme UBC9
UPR	Unfolded protein response
WIP1	Protein phosphatase 1D
WRN	Werner syndrome ATP-dependent helicase
XBP1	X-box-binding protein 1
XPB	General transcription and DNA repair factor IIH helicase subunit XPB
XPC	DNA repair protein complementing XP-C
XPD	General transcription and DNA repair factor IIH helicase subunit XPD
XRCC4	X-ray repair cross-complementing protein 4
